# Polymorphism in the EREG gene confers susceptibility to tuberculosis

**DOI:** 10.1186/s12881-018-0729-z

**Published:** 2019-01-11

**Authors:** Wen Cao, Liu-lin Luo, Wei-wei Chen, Li Liang, Ran-ran Zhang, Yan-lin Zhao, Jin Chen, Jun Yue

**Affiliations:** 1 Shanghai Key Laboratory of Mycobacterium Tuberculosis, Shanghai Pulmonary Hospital Affiliated to Tongji University School of Medicine, Shanghai, People’s Republic of China; 2 National Center for Tuberculosis Control and Prevention, Chinese Center for Disease Control and Prevention, Beijing, People’s Republic of China

**Keywords:** Tuberculosis, Epiregulin, Single nucleotide polymorphism, Susceptibility

## Abstract

**Background:**

Host genetic factors affect the immune response to *Mycobacterium tuberculosis* (*Mtb*) infection as well as the progression of the disease. Epiregulin (EREG) belongs to the epidermal growth factor (EGF) family, which binds to the epidermal growth factor receptor (EGFR) to regulate the immune response of the host during infections. Our study aimed to compare EREG levels in tuberculosis (TB) patients and healthy controls and assess whether polymorphisms in *EREG* increase the risk of TB.

**Methods:**

We used ELISA to determine the plasma EREG level from 30 healthy controls and 50 tuberculosis patients. By evaluating the *EREG* gene from 624 TB patients and 600 healthy controls, we determined the allelic and genotypic frequencies for association with susceptibility to TB infections in this group.

**Results:**

This paper shows that the pulmonary tuberculosis (PTB) and extrapulmonary tuberculosis (EPTB) groups showed a significantly higher plasma EREG level **(**1014 ± 733.9 pg/ml, 700.2 ± 676.6 pg/ml, respectively) than the healthy controls (277 ± 105.4 pg/ml). The rs2367707 polymorphism was associated with a higher risk of PTB and EPTB (*P* = 0.00051, *P* = 0.0012). Analyses of haplotype frequencies found that people with the haplotype CACAT had a higher risk of PTB and EPTB (*P* = 0.00031, OR = 1.43; *P* = 0.000053, OR = 1.65). Moreover, the rs6446993 polymorphism of the *EREG* gene was found to be associated with EPTB (*P* = 0.00087, OR = 1.54; 95% CI = 1.23–1.94).

**Conclusions:**

Compared to that of healthy controls, the level of EREG in the plasma of TB patients increased significantly. Based on these data, we demonstrated that *EREG* polymorphisms are genetic factors for susceptibility to TB and various forms of TB.

## Background

Tuberculosis (TB) is an infectious disease caused by *Mycobacterium tuberculosis (Mtb)* and has been a leading cause of human mortality worldwide over many decades, with an estimated global burden of 1.4 million TB deaths and 10.4 million new TB cases in 2017 [1]. Macrophages play a crucial role in the immune system by ingesting and degrading invading *Mtb*; they are a link between the inflammatory response and the adaptive immune response [2]. Host genetic factors affect the immune response to *Mtb* infection and the occurrence and development of TB. Candidate gene and genome-wide association (GWAS) research has studied the relationship between the human genetic background and susceptibility to TB, but the mechanism is unknown [3, 4]. Describing the interplay between host genetics and *Mtb* may provide insight into the occurrence, progression and control of the disease.

Epiregulin (EREG) belongs to the epidermal growth factor (EGF) family, whose members bind to the epidermal growth factor receptor (EGFR) or ErbB4 to generate signals for proliferation, migration, differentiation, cytokine secretion and innate immunity [5]. Compared with the expression in PTB and LTB patients, the expression of EREG in macrophages from patients with TBM increased [6]. Macrophages express EREG to modulate the host immune response to TLR ligands. The expression of EREG in the lungs of mice infected with *Mtb* was also significantly increased [7]. Recent data have suggested that EREG expression is also induced in monocytes after stimulating with *Mtb* and TLR4 and TLR2/1/6 ligands. In murine macrophages, EREG expression induced by *Mtb* is TLR2- and MYD88- dependent. Taken together, these studies demonstrate that EREG plays a functional role in TB pathogenesis and innate immunity [8]. EREG exists in two forms: a membrane-bound form and mature secreted form. The membrane-bound form regulates cytokine production in macrophage [9]. Compared to the cytokine levels of wild-type mice, IL-6 and TNF-α levels were lower in peritoneal macrophages (PM) from *EREG* knockout mice stimulated with lipopolysaccharides (LPS) and peptidoglycan (PGN). By downregulating IL-18, soluble EREG played a role in modulating the inflammatory pathway [10]. These data suggest that EREG is crucial for the control of *Mtb* infection. Therefore, we hypothesized that polymorphisms of the *EREG* gene may influence *Mtb* infection in humans.

In this paper, our goal was to determine *EREG* gene SNPs and the level of EREG in the plasma of TB patients compared to healthy controls.

## Materials and methods

### Subjects

In this case-control study, 1224 subjects were recruited: 600 healthy controls (HC), 424 pulmonary TB patients (PTB) and 200 extra-pulmonary TB patients (EPTB). All volunteers were enlisted from the Shanghai Pulmonary Hospital. Members of the control population were > 18 years of age and attested to no history of TB; their PPD tests and QFT tests were negative, and no evidence of prior TB presented in the chest radiographies. There were 340 males and 260 females, and the mean age was 34.66 ± 9.70. *Mtb* infections were confirmed in the TB patients included according to evidence of positive sputum smears and cultures, as well as clinical and radiography features. In the PTB groups, there were 250 males and 174 females, and the mean age was 35.44 ± 13.65. In the EPTB groups, there were 121 males and 79 females, and the mean age was 35.63 ± 17.22; there were 13 patients with intestinal tuberculosis, 10 patients with bone tuberculosis, 16 patients with lymph node tuberculosis, 60 patients with meningeal tuberculosis, 26 patients with genital tuberculosis, 64 patients with pleurisy tuberculosis, and 11 patients with renal tuberculosis, as shown in Table 1.

**Table 1:** Clinical characteristics of individuals stratified according to differences in infection locations

Subgroup	Number	Male	Female	*P*	Age ^c^	P*
Control	600	340 (56.7%)	260 (43.3%)		34.66 ± 9.70	
PTB ^a^	424	250 (59.0%)	174 (41.0%)	0.46^a^	35.44 ±13.65	0.26^a^
EPTB ^b^	200	121 (60.5%)	79 (39.5%)	0.34^b^	35.63 ±17.22	0.33^b^
Intestine TB	13	6	7		40.85 ±18.87	
Bone TB	10	4	6		36.30 ±10.37	
LNTB	16	4	12		44.25 ±15.64	
TB Meningitis	60	40	20		33.68 ±18.04	
Genital TB	26	18	8		41.19 ±14.18	
Pleurisy TB	64	42	22		31.81 ±16.39	
Renal TB	11	7	4		35.91 ±23.23	

^a^ *PTB* pulmonary tuberculosis patients

^b^ *EPTB* extra-pulmonary tuberculosis patients

^c^ Age (years) =Mean ±SD

*p*-value calculated by χ^2^ test for gender between TB patients and Controls

*P-value calculated by *t*-test for age between TB patients and Controls

### Ethics statement

All subjects volunteered for the study and signed informed consent forms. In addition, the Ethics Committee of Shanghai Pulmonary Hospital affiliated with Tongji University School of Medicine approved the study.

### Determination of plasma EREG levels

Thirty healthy controls and 50 TB patients, who were not selected from the genotyped individuals, were picked randomly to have their plasma separated from 200 μl EDTA anticoagulated blood by centrifugation. Following the manufacturer’s protocol, ELISA kits (R&D systems, MN, USA) were used for detecting EREG levels.

### Selection of DNA variants for *EREG* genotyping

We selected 5 SNPs from *EREG* (rs10518126, rs2367707, rs3806794, rs6446993, rs6836436), and the tag SNPs were chosen from the 1000 Genomes Project Phage3. The general rule for selecting tagged SNPs were an R^2^ linkage disequilibrium of > 0.8 and a minor allelic frequency of > 0.1. PCR primers were designed with Primer 3 software (http://bioinfo.ut.ee/primer3-0.4.0/). The genetic information and the primers are shown in Table 2.

**Table 2:** Single nucleotide polymorphisms (SNPs) of *EREG* gene

SNP	Gene	Chr	SNP property	PCR primer^a^	Alleles
rs10518126	EREG	4	Intron1	F:CACAAGACTGCCTTTCCACCATAA	C/T
				R:CCTCTCAGGGCCAATTTGAGGA	
rs2367707	EREG	4	exon4	F:TGGGTTATACTGGTGTCCGATGTG	A/G
				R:AATATGTGGAACCGACGACTGTGA	
rs3806794	EREG	4	5'-flanking	F:CAGGGGAGCGACAGGATTAAGG	A/C
				R:TGAGACCCCTGCTTCCAATGTG	
rs6446993	EREG	4	intron1	F:TGCACTCCATAGCCTGTGATCG	A/T
				R:CCTCATTCCAACTCCAGGGAGAA	
rs6836436	EREG	4	exon1	F:GTTCGCAGCACCAGACAGTTGA	G/T
				R:GCACAGAGCATCTCCATCCTCCT	

^a^ *F* forward primer, *R* reverse primer

Following the manufacturer’s protocol, we extracted genomic DNA from 200 μl EDTA anticoagulated blood with a QIAamp DNA Blood Midi Kit (Qiagen, Hilden, Germany). A SNaPshot® Kit was used to genotype the SNPs of the *EREG* gene.

We amplified all of the fragments by a Multiplex PCR reaction. The amplification system included 1 × HotStar Taq buffer, 0.3 mM dNTP, 3.0 mM Mg^2+^, 1 U HotStar Taq polymerase, 0.1 μM primer and 1 μl of DNA template. The reaction program carried out was as follows: 95 °C for 2 min; 11 cycles of 94 °C for 20 s, 65 °C ± 0.5 °C /cycle for 40 s, and 72 °C for 1 min 30 s; 26 cycles of 94 °C for 20 s, 59 °C for 30 s, and 72 °C for 1 min 30 s; and finally 72 °C for 2 min. Then, we added 5 U of shrimp alkaline phosphataseand 2 U of Exonuclease I to 15 μl of PCR product for purification. Next, we incubated the mixture at 37 °C for 60 min and then at 75 °C for 15 min. We performed the extension reaction with a SNaPshot® Kit, and a SNaPshot genotyping system was optimized sufficiently and validated by sequencing. The extension primers used to detect polymorphisms in the EREG genes were rs2367707SF (TTTTTTTTCCGTCCACCAACCTTTAAGCAAAGA), rs10518126SR (TTTTTTTTTTTGGGCCACTTTATAGAATTTGGAAATA), rs6836436SF (TTTTTTTTTTTTTTTTTTTTTTTTTTTTTTTCCCTTCTAGGCTGACAGCCGC), rs3806794SF (TTTTTTTTTTTTTTTTTTTTTTTAATAACAGGAATTTTCTTCACAATGACT), and rs6446993SR (TTTTTTTTTTTTTTTTTTTTTTTTTTTTTTTTAACAGAAAGGCTATTTAGAAAA). The extension reaction mixture was as follows: 5 μl of SNaPshot mix, 2 μl of the extension primer mix, 2 μl of purified PCR product and 2 μl of ultrapure water. The extension procedure was as follows: 96 °C for 1 min and 28 cycles of 96 °C for 10 s, 52 °C for 5 s, and 60 °C for 30 s. Then, we added 1 U of SAP to the extension products and incubated the mixture at 37 °C for 60 min followed by 75 °C for 15 min to purify the extension products. Then, we denatured the mixture using 1 μl of purified extension product or 0.5 μl of purified linkage product, 9 μl of HiDi formamide and 0.5 μl of the Liz120 size standard (Applied Biosystems, Foster City, USA) at 95 °C for 5 min. Finally, the mixture was loaded on an ABI 3730XL DNA Analyser (Applied Biosystems, Foster City, USA), and the results were analyzed using GeneMapper.

### Statistical analysis

The EREG levels were represented as the mean ± standard deviation, and the data between healthy controls and TB patients were analyzed with the t-test using SPSS 22.0. For each individual genotype, the OR and 95% confidence interval (CI) were estimated using logistic regression models adjusted for gender and age.

The Chi-squared test was used to evaluate whether the genotypic frequency in the population was consistent with the HWE. Data were confirmed to be within population equilibrium by calculating the HWE. We performed Chi-squared and Fisher’s exact tests to compare allelic and genotypic frequencies. The haplotypes and linkage disequilibrium in the HC and TB patients were analyzed by the SHEsis system. After Bonferroni correction, P*-values were five times the observed *P*-values, and *P* values < 0.05 were considered statistically significant.

## Results

### The EREG plasma levels from 30 healthy controls and 50 patients with tuberculosis

**Fig. 1:**
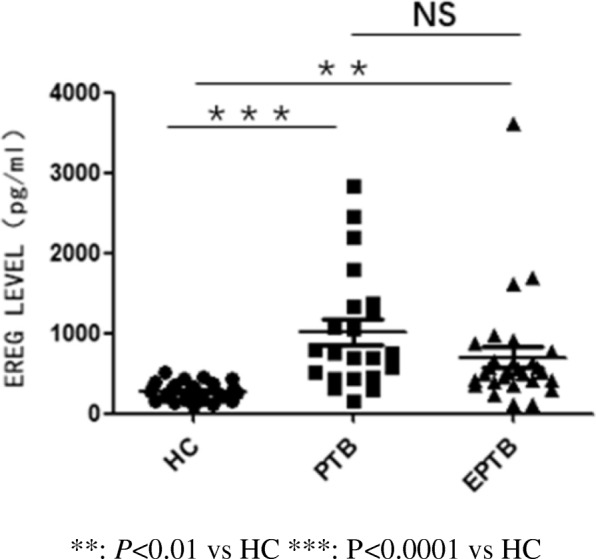
The EREG level were measured by ELISA kit. HC: healthy controls PTB: pulmonary tuberculosis EPTB: extra-pulmonary tuberculosis. **: *P* < 0.01 vs HC ***: *P* < 0.0001 vs HC

The plasma EREG levels (mean ± SD) were 1014 ± 733.9 pg/ml in PTB group and 700.2 ± 676.6 pg/ml in EPTB group and were significantly different from those of the healthy control group (277 ± 105.4 pg/ml) (Fig. 1).

### Data analyses of the *EREG* polymorphisms in PTB and HC patients

The allelic and genotypic frequencies of *EREG* polymorphisms in HC and PTB patients are shown in Table 3. We investigated the association of five *EREG* SNPs and PTB susceptibility using a case-control study. In the tested populations, all polymorphisms were consistent with Hardy-Weinberg equilibrium. Analyses of the distributions of PTB patients and healthy controls demonstrated that the *EREG* rs2367707 SNP contributed to susceptibility to PTB. The frequencies of the A allele of rs2367707 were significantly higher in HC than in PTB patients (*P* = 0.00019, OR (95% CI) =1.49 (1.23–1.81)).

**Table 3:** Allele and genotypic frequencies of *EREG* SNPs in the PTB and controls

SNP	Allele/genotype	PTB (%)	HC (%)	*P*	OR[95%CI]a
rs10518126	C	774 (91.3)	1092 (91.0)	0.83	
	T	74 (8.7)	108 (9.0)		
	C/C	352 (83.0)	492 (82.0)	0.20	
	C/T	70 (16.5)	108 (18.0)		
	T/T	2 (0.5)	0 (0.0)		
rs2367707	A	292 (34.4)	312 (26.0)	0.00019*	1.49 (1.23-1.81)
	G	556 (65.6)	888 (74.0)		
	A/A	48 (11.3)	48 (8.0)	0.00051*	1.86 (1.19-2.88)
	A/G	196 (46.2)	216 (36.0)		
	G/G	180 (42.5)	336 (56.0)		
rs3806794	A	88 (10.4)	112 (9.3)	0.43	
	C	760 (89.6)	1088 (90.7)		
	A/A	6 (1.4)	9 (1.5)	0.63	
	A/C	76 (17.9)	94 (15.7)		
	C/C	342 (80.7)	497 (82.8)		
rs6446993	A	364 (42.9)	486 (40.5)	0.27	
	T	484 (57.1)	714 (59.5)		
	A/A	76 (17.9)	108 (18.0)	0.22	
	A/T	212 (50.0)	270 (45.0)		
	T/T	136 (32.1)	222 (37.0)		
rs6836436	G	76 (9.0)	126 (10.5)	0.25	
	T	772 (91.0)	1074 (89.5)		
	G/G	2 (0.5)	6 (1.0)	0.44	
	G/T	72 (17.0)	114 (19.0)		
	T/T	350 (82.5)	480 (80.0)		

* *P* indicates a significant association after Bonferroni correction for multiple testing at significance level α=0.05

^a^Adjusted for age and gender from a logistics regression model

Comparing the genotypic frequencies between healthy controls and PTB patients, we found that rs2367707 A/G was more common in PTB patients (*P* = 0.00051). For the distribution of the other four *EREG* SNPs between PTB patients and controls, no significant differences were observed.

### Data analyses of the *EREG* polymorphisms in EPTB patients and HC

The allelic and genotypic frequencies of *EREG* polymorphism in EPTB patients and healthy controls are indicated in Table 4. Analyses of the distributions of EPTB patients and healthy controls demonstrated that the *EREG* rs2367707 and rs6446993 SNPs contributed to susceptibility to EPTB. The frequencies of the A allele of rs2367707 and the T allele of rs6446993 were significantly higher in healthy controls than in EPTB patients (*P* = 0.00029, OR (95% CI) =1.64 (1.28–2.08); *P* = 0.00087, OR (95% CI) =1.54 (1.23–1.94)).

**Table 4:** Allele and genotypic frequencies of *EREG* SNPs in the EPTB and controls

SNP	Allele/genotype	EPTB (%)	HC (%)	*P*	OR[95%CI]^a^
rs10518126	C	353 (88.2)	1092 (91.0)	0.11	
	T	47 (11.7)	108 (9.0)		
	C/C	158 (79.0)	492 (82.0)	0.0026*	1.08(0.71-1.64)
	C/T	37 (18.5)	108 (18.0)		
	T/T	5 (2.5)	0 (0.0)		
rs2367707	A	146 (36.5)	312 (26.0)	0.00029*	1.64 (1.29-2.08)
	G	254 (63.5)	888 (74.0)		
	A/A	25 (12.5)	48 (8.0)	0.0012*	2.22 (1.29-3.83)
	A/G	96 (48.0)	216 (36.0)		
	G/G	79 (39.5)	336 (56.0)		
rs3806794	A	46 (11.5)	112 (9.3)	0.21	
	C	354 (88.5)	1088 (90.7)		
	A/A	7 (3.5)	9 (1.5)	0.21	
	A/C	32 (16.0)	94 (15.7)		
	C/C	161 (80.5)	497 (82.8)		
rs6446993	A	205 (51.2)	486 (40.5)	0.00087*	1.54 (1.23-1.94)
	T	195 (48.7)	714 (59.5)		
	A/A	55 (27.5)	108 (18.0)	0.0069*	0.68 (0.45-1.02)
	A/T	95 (47.5)	270 (45.0)		
	T/T	50 (25.0)	222 (37.0)		
rs6836436	G	50 (12.5)	126 (10.5)	0.27	
	T	350 (87.5)	1074 (89.5)		
	G/G	5 (2.5)	6 (1.0)	0.27	
	G/T	40 (20.0)	114 (19.0)		
	T/T	155 (77.5)	480 (80.0)		

* *P* indicates a significant association after Bonferroni correction for multiple testing at significance level ɑ=0.05

^a^ Adjusted for age and gender from a logistics regression model

Comparing genotypic frequencies between healthy controls and EPTB patients, we found that rs2367707 A/G and rs6446993 A/T were more common in EPTB patients (*P* = 0.0012, *P* = 0.0069).

### Haplotype and linkage disequilibrium (LD) analyses

The haplotype data between the two disease forms are shown in Table 5 and Table 6. We identified 11 haplotypes with SHEsis, and seven were excluded with a frequency < 0.03. Haplotype analysis showed that the *EREG* CACAT and CGCTT haplotypes were significantly related to PTB and EPTB and that the CACAT haplotype was a significantly “beneficial” haplotype (*P* = 0.00031 for PTB, *P* = 0.000053 for EPTB); the other two haplotypes showed no significant association.

**Table 5:** Estimated frequencies of haplotypes consisting of *EREG* SNPs in PTB and controls

Haplotype	PTB (%)^a^	Control (%)^a^	χ2	*P*	OR[95%CI]
CACAT	279.88 (33.0)	303.06 (25.3)	13.02	0.00031	1.43 (1.18-1.74)
CGATT	84.46 (10.0)	104.24 (8.7)	0.75	0.39	1.14 (0.85-1.54)
CGCTT	387.42 (45.7)	607.71 (50.6)	6.77	0.0093	0.79 (0.66-0.94)
TGCAG	73.76 (8.7)	101.12 (8.5)	0.011	0.92	1.017 (0.74-1.39)

^a^ Frequencies <0.03 were excluded from the analysis

Global p<0.01

The order of the SNPs in the haplotype is rs10518126 rs2367707 rs3806794 rs6446993 rs6836436

**Table 6:** Estimated frequencies of haplotypes consisting of *EREG* SNPs in EPTB and controls

Haplotype	EPTB (%)^a^	Control (%)^a^	χ2	P	OR[95%CI]
CACAT	145.99 (36.5)	303.06 (25.3)	16.35	0.000053	1.65 (1.29-2.10)
CGATT	45.99 (11.5)	104.24 (8.7)	2.29	0.13	1.33 (0.92-1.92)
CGCTT	149.01 (37.3)	607.71 (50.6)	26.09	3.32×10 ^-7^	0.55 (0.43-0.69)
TGCAG	47.00 (11.7)	101.12 (8.4)	3.35	0.070	1.41 (0.98-2.03)

^a^ Frequencies <0.03 were excluded from the analysis

Global p<0.01

The order of the SNPs in the haplotype is rs10518126 rs2367707 rs3806794 rs6446993 rs6836436

**Fig. 2:**
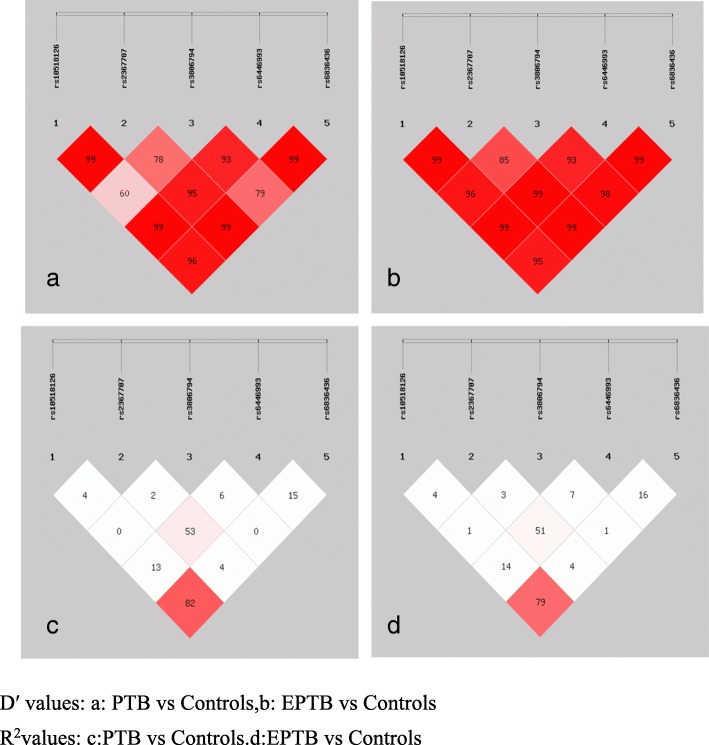
Linkage disequilibrium (LD) structure of SNPs in EREG. D′ values (%) and r^2^ are indicated on squares. Pairwise values are color coded: high values are dark, low values are light. All values were generated using SHEsis software. D′ values: **a** PTB vs Controls, **b** EPTB vs Controls. R^2^values: **c** PTB vs Controls. **d** EPTB vs Controls

We assessed the LD among SNPs with both D’ and r^2^. The LD structure constructed with five SNPs (rs10518126, rs237707, rs3806794, rs6446993, and rs6836436) is shown in Fig. 2. The degree of LD among the five SNPs was relatively high.

## Discussion

Tuberculosis is an infectious disease that can invade multiple systems, such as the pulmonary, intestinal, renal and central nervous systems (CNS). Numerous immune signaling pathways, inflammation responses and cell types (e.g., macrophage, dendritic cells, T cells) are involved in the initiation, progression and pathogenesis of tuberculosis. Macrophages express Toll-like receptors (TLRs), which can recognize pathogen-associated molecular patterns (PAMPs) of *Mtb* and regulate the production of immune-associated cytokines [11]. EREG acts as an epidermal growth factor receptor ligand that exhibits a regulatory property by inhibiting the growth of epithelial cells [12] as well as regulating inflammation [13] and the immune response [10] during infection. NTT Thuong [8] showed that the expression of EREG depends on TLR activation, may regulate TLR-associated signaling of macrophages and is a highly induced TLR-dependent gene associated with risk for TB. Many studies showed that EREG may impact bacterial pathogenesis and disease outcome through a biosynthesis and regulatory role in innate immune defenses. Previous studies have demonstrated that EREG played a potential role in various bacterial infections, such as *Mycoplasma pneumonia* [14], *Streptococcus suis* [15] and *Mycobacterium tuberculosis* [7], but because of the different interactions between the bacterial and the host immune response, the phenotypic effects on different infections were also different.

Associations of polymorphisms in *EREG* and susceptibility to TB have been reported in a variety of populations. In populations from Guinea-Bissau and Gambia, the *EREG* SNP rs1563826 was almost significant [16]. Another case-control study showed evidence for an association between rs7675690 in *EREG* and pulmonary TB, particularly meningeal tuberculosis [8]. In our study, we assessed EREG plasma levels, and we analyzed a Chinese population (*n* = 600 controls and *n* = 624 cases) for polymorphisms in *EREG* and whether previously described associations with TB in other populations would be replicated in a Chinese population. We evaluated five SNPs of *EREG* for associations with TB and different clinical forms. Our hypothesis was that SNPs of *EREG* would confer susceptibility to TB.

Our data showed significantly higher plasma concentrations of EREG in TB patients compared to the healthy controls. EREG has been shown to be upregulated on macrophages in TB; this finding was consistent with our results. Moreover, we found an association of an *EREG* polymorphism with TB. The allelic and genotypic frequencies of the *EREG* gene differed between the two groups. The G allele of rs2367707 was more universal in the controls than in PTB and EPTB patients. Moreover, the rs2367707 A/G genotype showed a high risk of association with TB. In our study, the distribution of haplotypes between controls and patients was significantly different, particularly for CACAT and CGCTT. *EREG* SNPs within PTB and EPTB were further examined. The A allele and A/G genotype of rs2367707 showed significant effects on PTB. The alleles and genotypes of the *EREG* SNPs rs2367707 and rs6446993 were strongly associated with EPTB. A candidate gene study found that the *EREG* rs2367707 was a potential genetic risk factor for chronic temporomandibular disorders (TMD) [17], but the mechanism was unknown. These data showed that rs2367707 in exon4 of *EREG* was related to disease, and further investigations should concentrate on its function in disease. In addition, we showed that rs6446993 located in the intron may affect the activity of *EREG* because SNPs located in introns may influence RNA splicing by altering the formation of splicesomes, leading to changes in protein structure and folding. Thus, it is a novel target for therapy.

EREG has been identified as a regulator of inflammation and is associated with disease; its levels are higher in patients with chronic inflammatory disorders [18]. Previous studies have shown that EREG expression is induced in human macrophages and monocytes after *Mtb* stimulation [19]. EREG is expressed not only in macrophages but also in keratinocytes, and a previous study revealed that *EREG*-deficient mice develop chronic dermatitis, a major cause of skin inflammation [20]. In recent years, many researchers have demonstrated the effect of EREG in controlling inflammation on colon disease [21], rhinosinusitis [22] and cancer [23]. Several previous experiments have concentrated on the association between *EREG* SNPs and various diseases. Takeru Wakatsukiet al. [24] identified SNPs within the genomic regions of *EREG* in gastric cancer for the first time and demonstrated an association of those SNPs with the 3-year RFS (recurrence free) and 3-year overall survival rates. In a study of Behçet’s disease (BD) with a case-control study of 976 Iranian patients and 839 healthy controls, a new SNP, rs6845297, located downstream of *EREG*, was associated with BD [25].

In conclusion, our paper suggests genetic variants of *EREG* confer susceptibility to TB. We demonstrated that *EREG* polymorphisms may affect the development of TB, which suggests that *EREG* may be a potential target for TB treatment. However, these results should be conducted with larger numbers of subjects and subjects from other geographic locations. Moreover, experimental research of how the *EREG* polymorphism affects the molecular mechanism of the occurrence and development of TB is needed.

## Conclusions

In summary, we suggest that *EREG* gene polymorphism confers susceptibility to pulmonary tuberculosis and extra-pulmonary tuberculosis. However, these results should be conducted with larger and geographically broader population samples. Moreover, experimental research on the molecular mechanism of how the *EREG* polymorphism affects the occurrence and development of TB is needed.

## Abbreviations

EPTB: Extrapulmonary tuberculosis
EREG: Epiregulin
LD: Linkage disequilibrium
*Mtb*: *Mycobacterium tuberculosis*
PTB: Pulmonary tuberculosis
SNP: Single nucleotide polymorphism
TLRs: Toll-like receptors
